# Supplementary material for: The MultimorbiditY COllaborative Medication Review And DEcision Making (MyComrade) study: a protocol for a cross-border pilot cluster randomised controlled trial
Source: Pilot Feasibility Stud. 2022 Mar 28;8:73. doi: 10.1186/s40814-022-01018-y (PMC8958932; doi:10.1186/s40814-022-01018-y)
Supplement: Supplementary file 2 — Additional file 2. The TIDieR (Template for Intervention Description and Replication) Checklist. [file 40814_2022_1018_MOESM2_ESM.docx]

**The TIDieR (Template for Intervention Description and Replication) Checklist**

**Supplementary data for: The MultimorbiditY COllaborative Medication Review And DEcision Making (MyComrade) study: A protocol for a cross-border pilot cluster randomised controlled trial.**

|  | Item | Where located |
| --- | --- | --- |
|  | **BRIEF NAME** |  |
| 1. | MultimorbiditY COllaborative Medication Review And DEcision Making (MyComrade) | Page 1 |
|  | **WHY** |  |
| 2. | We used the results of a systematic review and qualitative interview together with the Capability- Opportunity-Motivation-Behaviour (COM-B) model of behaviour, the Behaviour Change Wheel approach to intervention development and the Behaviour Change Technique (BCT) taxonomy to develop this intervention specifically to facilitate the conduct of active medication review. Based on the findings of a feasibility study, one additional BCT was added to address the need for additional supports to complete medication reviews. | Page 5 and <http://www.implementationscience.com/content/10/1/132>; https://pilotfeasibilitystudies.biomedcentral.com/articles/10.1186/s40814-017-0129-8 |
|  | **WHAT** |  |
| 3. | Participants were pairs of General Practitioners (GPs) in the Republic of Ireland (ROI), and a GP and Practice-Based Pharmacist (PBP) in Norther Ireland (NI). GPs/GP and PBPs received a brief information session and were provided with instructions on how to implement the intervention. The instructions detailed the six behaviour change techniques included in the intervention. They were also provided with a booklet containing copies of a medication review checklist. This checklist was a modified version of the NO TEARS tool for medication review. | GP participant information leaflet  <https://drive.google.com/file/d/1-3S2nK5HZjADEW04n01NCny-oJxdquvL/view?usp=sharing>  Instructions for participating GPs/PBPs  <https://drive.google.com/file/d/12sjxYC9sqsq4NAdvBNZz0GUSnenGKPCT/view?usp=sharing>  Medication review checklist  <https://drive.google.com/file/d/1_Y4QczlLgLykiDSCHc2jJpfHVvI3fMYe/view?usp=sharing> |
| 4. | Each pair of GPs or GP and PBP was asked to conduct 20 medication reviews using the MyComrade approach. Patients who were prescribed 10 or more medications were recruited from each practice. | Page 9-13 |
|  | **WHO PROVIDED** |  |
| 5. | Only practicing GPs/PBP implemented the intervention | Page 13 |
|  | **HOW** |  |
| 6. | GPs or GP and PBP implemented the intervention in pairs. | Page 13 |
|  | **WHERE** |  |
| 7. | The intervention was implemented in the GP practice. The participating GPs, or GP and PBP were asked to come up with an action plan in which they would specify when and where they would conduct the reviews. | Page 13 |
|  | **WHEN and HOW MUCH** |  |
| 8. | Each pair of GPs, or GP and PBP was asked to conduct 20 medication reviews using the MyComrade approach. Patients were recruited from each practice who were prescribed 10 or more medications. They were asked to complete the reviews within a four month interval | Page 9-13 |
|  | **TAILORING** |  |
| 9. | Participating GPs, or GP and PBP were advised that they could adapt the action plan (when, where, how many patients to review in one sitting etc.) to suit their own practice. This adaption was captured in qualitative interviews. | Page 11, 18 and participant information sheets as above. |
|  | **MODIFICATIONS** |  |
| 10.^ǂ^ | As described above, the original intervention was modified based on findings from a feasibility study. Further modifications were made to account for contextual differences between the two jurisdictions involved in the trial – principally that the reviews were conducted by GP pairs in ROI and GP-PBP pairs in NI. | Page 14 and  https://pilotfeasibilitystudies.biomedcentral.com/articles/10.1186/s40814-017-0129-8 |
|  | **HOW WELL** |  |
| 11. | Intervention adherence will be assessed in qualitative interviews. | Page 18 |
| 12.^ǂ^ | Observation or recording of implementation of the intervention was not performed. |  |

** **Authors** - use N/A if an item is not applicable for the intervention being described. **Reviewers** – use ‘?’ if information about the element is not reported/not sufficiently reported.

† If the information is not provided in the primary paper, give details of where this information is available. This may include locations such as a published protocol or other published papers (provide citation details) or a website (provide the URL).

ǂ If completing the TIDieR checklist for a protocol, these items are not relevant to the protocol and cannot be described until the study is complete.

* We strongly recommend using this checklist in conjunction with the TIDieR guide (see *BMJ* 2014;348:g1687) which contains an explanation and elaboration for each item.

* The focus of TIDieR is on reporting details of the intervention elements (and where relevant, comparison elements) of a study. Other elements and methodological features of studies are covered by other reporting statements and checklists and have not been duplicated as part of the TIDieR checklist. When a **randomised trial** is being reported, the TIDieR checklist should be used in conjunction with the CONSORT statement (see [www.consort-statement.org](http://www.consort-statement.org)) as an extension of **Item 5 of the CONSORT 2010 Statement.** When a **clinical trial** **protocol** is being reported, the TIDieR checklist should be used in conjunction with the SPIRIT statement as an extension of **Item 11 of the SPIRIT 2013 Statement** (see [www.spirit-statement.org](http://www.spirit-statement.org)). For alternate study designs, TIDieR can be used in conjunction with the appropriate checklist for that study design (see [www.equator-network.org](http://www.equator-network.org)).
