# Supplementary material for: The MultimorbiditY COllaborative Medication Review And DEcision Making (MyComrade) study: a protocol for a cross-border pilot cluster randomised controlled trial
Source: Pilot Feasibility Stud. 2022 Mar 28;8:73. doi: 10.1186/s40814-022-01018-y (PMC8958932; doi:10.1186/s40814-022-01018-y)
Supplement: Supplementary file 3 — Additional file 3. Summary of data collection. [file 40814_2022_1018_MOESM3_ESM.docx]

Additional file 3. Summary of data collection

| **Item** | **Definition and measurement** | **Data source** |
| --- | --- | --- |
| **MC GP Practice Level Variables** | | |
| **GP Practice Demographics, Recruitment and Intervention** | | |
| Name & Location | Name & Location of GP Practice - NI or ROI | Practice Profile Questionnaire |
| Management Information System | GP practice patient management system (e.g. Socrates, HealthOne) |  |
| Setting | Demographic profile of the clients: If Urban, Rural, Mixed based |  |
| Staffing quota | Staff quota - Numbers of full & part time GPs, Trainee GPs, Practice Nurses, Practice Managers & administration staff |  |
| Staff Sessions | Number of staff sessions: GPs, Practice Nurses, Practice Managers & Admin |  |
| Training Practice | If practice is a GP training practice? Yes/No |  |
| Target Populations | Total number of patients registered with GP practice - ROI:(GMS, PP), NI:(NHS) |  |
| Repeat Prescribing Policy Document | Existence of a repeat medication prescribing policy? Length of time spent each day on repeat prescribing? How patients request their repeat prescription (e.g. telephone, e-mail)? Who generates repeat prescriptions (e.g. GP, Admin Staff)? If designated time assigned to management of repeat prescriptions?  If patient’s medical record routinely checked prior to signing the repeat prescription? If patients in receipt of repeat prescriptions get an annual face-to-face medication review? |  |
| Adverse Events Policy Document | If practice have a method of recording and reporting adverse event or near misses? |  |
| Database search | Number of potentially eligible patients on day of database search? | Database Search Results Record |
| Eligibility | Number of ineligible (detailing number of patients: prescribed<10 medications, <18yrs, undergoing terminal illness care, too frail, pregnant, limited capacity, deceased, left practice) and eligible patients | Database Search Results Record |
| Intervention | Completion of medication reviews Yes/No/Partial  Number of reviews completed (N)  Completed review upload Yes/No/Partial | Study Tracking Document |
|  | | |
| **MC Participant (patient) Level Variables - anonymised - collected at baseline** | | |
| Recruitment | Number of patients: invited, consented, eligible, ineligible and final recruitment and drop out with reasons | Study Tracking Document |
| **Participant (patient) Demographics** | | |
| Date of birth | Participant`s date of birth | Baseline questionnaire |
| Gender | Sex of Participant – Male/Female/Other - describe |  |
| Marital Status | Participant marital status at baseline |  |
| Native Language | Language spoken at home, if English - Yes/No |  |
| Education Attainment | Participant’s highest level of education attainment |  |
| Employment Status | Participant’s current job title or title of last paid job |  |
| Health Care Cover | Participant Health Cover – if Private, Public or Combined |  |
| Access to GP | Distance from participants home to GP practice & mode of travel |  |

| **MC Participant (patient) Level Variables - anonymised - collected at baseline, 4 and 8 months post randomisation** | | |
| --- | --- | --- |
| **Participant (patient) Healthcare Utilisation** | | |
| Participants (patient) Utilisation of GP, primary and (some) tertiary healthcare services | Number of: - GP face to face consultations at practice - GP telephone consultations  - GP patient management sessions - GP house calls to participant - GP out of hours visits to participant  - Repeat prescriptions without GP face to face consultation  - Practice Nurse face to face consultations at practice - Practice Nurse telephone consultations  - Practice Nurse management session  - Outpatient (OPD) visits  - Emergency department presentations (i.e. not admitted)  - Hospital admissions – day cases (i.e. discharged same day) - Hospital admissions – inpatient nights ( i.e. overnight stays) | Patient records - MyComrade Prescribing Outcomes Tool - Baseline, 4 & 8 months post randomisation |
| **Potentially Inappropriate Prescribing (PIP)** | | |
| Potential Inappropriate Prescribing | Evidence of any of the following PIP indicators and the number of each: - PIP 1: Having a medical history of ever or currently having a peptic ulcer? If yes, if currently prescribed a NSAID drug and/or PPI? - PIP 2: Having a medical history of asthma? If yes, having a medical history of CHD? If yes, if currently prescribed a Beta Blocker?  - PIP 3: If >/+ 75 years, if prescribed an angiotensin converting enzyme inhibitor or a loop diuretic long-term? If yes, have they an eGFR and/or ACR taken in last 6 months? - PIP 4: Having a prescription for the combined oral contraceptive pill? If yes, is there a medical history of venous or arterial thrombus?  - PIP 5: Prescribed Methotrexate in last 3 months? If yes, have they a Full Blood Count taken in last 3 months? - PIP 6: Prescribed Warfarin in last 3 months? If yes, have they an INR taken in last 3 months? - PIP 7: Prescribed Lithium in last 3 months? If yes, have they a lithium level taken in last 6 months? - PIP 8: Prescribed Amiodarone in last 6 month? If within 1 month, is dose >200mg/day? If yes, how much? Have they a TFT`s taken in last 6 months? | Patient records - Potential Inappropriate Prescribing (Avery, 2009) - MyComrade Prescribing Outcomes Tool - Baseline, 4 and 8 months post randomisation |
| **Medication prescribing history** | | |
| Participants (patient) medication prescribing history | Number of repeat medications prescribed | Patient records – Medication prescriptions - Baseline, 4 and 8 months post randomisation |
|  | Number of new repeat medications commenced and/or existing medications discontinued |  |
|  | Number of repeat medication de-prescribed |  |
| **Quality of Life** | | |
| Self-reported assessment on Quality of Life | Self-Reported assessment of own quality of life – statement that best describe health state in relation to: - Mobility,  - Performing self-care - Performing usual activities - Having pain & discomfort,  - Having anxiety and/or depression - Numeric rating health & well being | Patient Self-report questionnaire - EQ5D Tool (EuroQol Group, 1990) - Baseline, 4 and 8 months post randomisation |
| **Treatment burden** | | |
| Self-reported assessment on medical treatment burden | Self-reported assessment (1-6) on:  - Taking lots of medications - Remembering how and when to take medications  - Paying for prescriptions, over the counter medicines or equipment  - Collecting prescription medication  - Monitoring of medical conditions (e.g. blood pressure)  - Arranging appointments with health professionals - Seeing different healthcare professionals  - Attending appointments with health professionals  - Getting health care in the evenings at weekends  - Getting help from community services (e.g. Physiotherapists)  - Obtaining clear and up to date information about condition(s) - Making recommended lifestyle changes (e.g.. diet, exercise) - Having to rely on help from family and friends | Patient Self-report questionnaire - Multimorbidity Treatment Burden Questionnaire (Duncan et al., 2018) - Baseline, 4 and 8 months post randomisation |
