# Supplementary figures and images for: The MultimorbiditY COllaborative Medication Review And DEcision Making (MyComrade) study: a protocol for a cross-border pilot cluster randomised controlled trial
Source: Pilot Feasibility Stud. 2022 Mar 28;8:73. doi: 10.1186/s40814-022-01018-y (PMC8958932; doi:10.1186/s40814-022-01018-y)

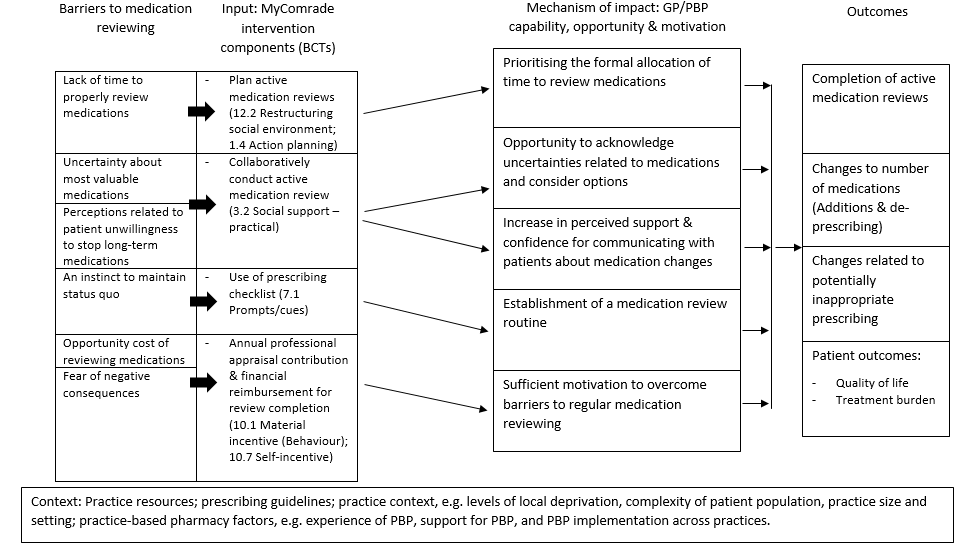

Supplement: Supplementary file 4 — Additional file 4. MyComrade Logic Model. [file 40814_2022_1018_MOESM4_ESM.docx]
